# Rare Earth-Based Compounds as Inhibitors of Hot-Corrosion Induced by Vanadium Salts

**DOI:** 10.3390/ma12223796

**Published:** 2019-11-19

**Authors:** N.S. Flores-Garcia, C.D. Arrieta-Gonzalez, J.J. Ramos-Hernandez, G.K. Pedraza-Basulto, J.G. Gonzalez-Rodriguez, J. Porcayo-Calderon, L. Martinez-Gomez

**Affiliations:** 1 Instituto de Ciencias Físicas, Universidad Nacional Autónoma de México, Avenida Universidad s/n, 62210 Cuernavaca, MOR, Mexicolmg.icf.unam@gmail.com (L.M.-G.); 2 Tecnológico Nacional de México - Instituto Tecnológico de Zacatepec, Calzada Instituto Tecnológico 27, 62780 Zacatepec, MOR, Mexico; cdaglez@gmail.com; 3 Facultad de Ingeniería, UNACAR, 24180 Ciudad del Carmen, CAM, Mexico; gabrielakarina.pedraza@gmail.com; 4 CIICAp, Universidad Autónoma del Estado de Morelos, Avenida Universidad 1001, 62209 Cuernavaca, MOR, Mexico; ggonzalez@uaem.mx; 5 Corrosion y Protección (CyP), Buffon 46, 11590 México City, CDMX, Mexico

**Keywords:** sodium metavanadate, hot corrosion, inhibitor, lanthanum vanadate, lanthanum oxide

## Abstract

In this study, the performance evaluation of lanthanum compounds as corrosion inhibitors of vanadium salts was performed. The inhibitors tested were lanthanum acetate and La_2_O_3_. The performance of the inhibitors was tested using sodium metavanadate (NaVO_3_) as a corrosive medium at 700, 800, and 900 °C. The corrosion inhibitory effect was evaluated on the corrosion process of 304H stainless steel. The corrosion rate of the steel was determined by the mass loss technique after 100 h of immersion in the corrosive salt with and without the addition of the corrosion inhibitor. The results show that lanthanum compounds act as corrosion inhibitors of vanadium salts. The inhibitory effect increases by increasing the concentration and tends to decrease when increasing the test temperature. Lanthanum compounds act as excellent corrosion inhibitors due to their ability to stabilize vanadium cations. Vanadium is stabilized by forming a new compound, lanthanum vanadate (LaVO_4_), with a melting point much higher than the compounds formed when Mg or Ni compounds are used as corrosion inhibitors.

## 1. Introduction

The use of low-quality fuel oil in many industrial processes has caused serious equipment availability problems due to premature failure of construction materials. These failures are associated with high corrosion rates caused by the inorganic components of low-grade fuels [1].

During the combustion of low quality fuels, the presence of elements such as S, Na, and V causes the formation of their oxides, which are transported by the combustion gases and condense (oxides of V and Na) onto metal surfaces, favoring the formation of corrosive species such as sodium sulfate, simple vanadates, and complex vanadates [2]. V and S are elements that are part of the macromolecules of crude oil, and the presence of Na is a consequence of contamination occurring during its extraction and transport [1].

In general, the wide range of vanadium salts that may exist in ash deposits is the result of the chemical reaction of two main compounds, namely, V_2_O_5_ and Na_2_SO_4_. However, depending on its molar ratio and temperature, the resulting vanadium salt may show different aggressiveness, as shown.

(1) $${\mathrm{xNa}}_{2}{\mathrm{SO}}_{4}+{\mathrm{yV}}_{2}O_{5}\to {\mathrm{xNa}}_{2}O\cdot {\mathrm{yV}}_{2}O_{5}+{\mathrm{xSO}}_{3}$$

In general, as shown in Table 1, increasing the V content reduces the melting point of the vanadium salt formed and consequently increases its corrosivity. This is due both to the increase in acidity and the oxidative capacity of the molten salt. The greater oxidizing power has been associated with the multivalent character of V, which increases the ionic conductivity (O^2−^) of the melt; this favors the accelerated oxidation process of the materials as well as the acidic dissolution of their protective oxides [3,4].

Due to the enormous temperature gradient existing in a boiler (from the combustion zone to the combustion gas exit through the chimney), chemical species deposited and formed on metal surfaces can be very diverse. The main corrosion problems of molten salts generally occur on thermal exchange surfaces (superheaters and reheaters) whose metal temperature is around 600 °C. However, the non-refrigerated elements (spacers, baffle screens, etc.) can reach metal temperatures greater than 600 °C, and therefore, their useful life is very short. This variety of environmental conditions makes the degradation mechanism very complex.

Vanadium salts are considered the most corrosive species of molten salts. Ash deposits in the molten state are ionic electrolytes. They have a high absorption capacity for oxygen and other oxidizing species present in the flue gases, which causes the accelerated attack of the metal surfaces. Due to their acidity, they are capable of dissolving virtually all protective oxides developed on metal surfaces [5]. Many of the materials used in the heat exchange surfaces base their corrosion resistance on the development of a Cr-rich protective oxide; however, its protective capacity decreases due to its high solubility in molten salts rich in V [6]. When adjusting the boiler’s operating conditions (reducing the oxidation state of V) or changing the type of fuel oil (for a lower concentration of inorganic matter) is not a viable alternative, then the main options to reduce corrosion problems may be the use of materials with greater corrosion resistance, the use of protective metal coatings, or the use of corrosion inhibitors.

A decrease in the concentration of V, both in ash and combustion gases, shifts the chemical equilibrium towards basic conditions, where the aggressiveness of vanadium salts is low [6]. In addition, a reduction in the availability of V may be possible through its complexation with another element that allows the formation of highly stable refractory vanadates (high melting point).

In this regard, the literature has cited many efforts to develop corrosion inhibitors of molten vanadium salts. The main objective of corrosion inhibitors is to stabilize V by trapping it in new compounds with a high melting point and low chemical reactivity. To date, the most commonly used corrosion inhibitor is based on magnesium compounds that, when reacted with vanadium salts, form the refractory compound Mg_3_(VO_4_)_2_ (melting point of 1074 °C) [7,8,9,10,11]. However, its inhibition capacity is compromised if the S content of the fuel is high [12,13]. This leads to the Mg compounds formed not being the expected refractory compounds. Other proposals are based on the use of NiO, which is capable of reacting with vanadium salts, forming refractory compounds (Ni_3_V_2_O_8_) with a higher melting point (1310 °C) than those obtained if Mg compounds are used as inhibitors [9,11]. These studies are consistent with those that indicate that metallic Ni shows a high resistance to corrosion by V salts due to the formation of a protective barrier of Ni_3_V_2_O_8_ on its surface [14,15].

Because no new proposals have been reported to solve corrosion problems caused by vanadium salts in the molten state, this study proposes the use of rare-earth-based compounds as potential corrosion inhibitors. The use of two La compounds is considered, and their inhibition capacity is evaluated on the corrosion process of 304H stainless steel in NaVO_3_ at different temperatures.

## 2. Materials and Methods

### 2.1. Materials

As a test material, a steel commonly used in boiler heat exchangers that burn low-quality fuel oils, namely 304H stainless steel, was used. Samples of 304H steel with dimensions of 10 × 10 × 3 mm were abraded with silicon carbide abrasive paper up to 600 grit and subsequently washed with distilled water and acetone. The dimensions of the metal samples were taken with an accuracy of 0.1 mm and weighed on an analytical balance with an accuracy of 0.1 mg.

### 2.2. Corrosive Medium

As a corrosive medium, sodium metavanadate (NaVO_3_) was used. This vanadium salt is used in corrosion studies to analyze the effect of vanadium on the corrosion process of materials. The amount of corrosive salt used was 500 mg/cm^2^ with respect to the area of the metal samples.

### 2.3. Corrosion Inhibitors

This study proposes the use of two lanthanum compounds as potential inhibitors of corrosion caused by molten vanadium salts. The lanthanum compounds evaluated are lanthanum oxide (La_2_O_3_) and hydrated lanthanum acetate (La(CH_3_COO)_3_·1.5H_2_O), for simplicity, La(Ac)_3_. La_2_O_3_ was considered as an alternative corrosion inhibitor because it can be injected directly at any point in the boiler and La(Ac)_3_ as an alternative corrosion inhibitor that can be mixed with the fuel. Corrosion tests were performed with the addition of 0%, 5%, and 10% inhibitor with respect to the mass of the corrosive salt. 

### 2.4. Corrosion Assays

Corrosion tests were performed using the mass loss technique. For this, samples of 304H steel were placed inside alumina crucibles together with the corrosive medium. Corrosion tests were performed in an electric oven in static air conditions for 100 h. The test temperatures were 700, 800, and 900 °C. The tests were carried out in quadruplicate; at the end of each test, three samples were used to determine the loss of mass, and the fourth sample was encapsulated in epoxy resin for surface preparation and analysis by scanning electron microscopy and energy dispersive spectroscopy (EDS).

In order to determine the mass loss of the corroded samples, the corrosion product-layer was removed, and the metal surface was cleaned according to the procedures suggested in ASTM G1 (AMERICAN SOCIETY FOR TESTING AND MATERIALS). In summary, the procedure used consisted of alternating mechanical and chemical cleaning until the total elimination of corrosion products with a minimum removal of sound metal. The mechanical cleaning consisted of brushing with a soft bristle brush. Chemical cleaning consisted of the removal of the layers of corrosion products by dissolution in a chemical solution at 60 °C. The chemical solution used consisted of an aqueous solution with 10 g of citric acid, 5 mL of sulfuric acid, and 0.02 g of diorthotolyl thiourea per 100 mL of solution. At each stage of the cleaning cycle, the samples were washed with distilled water and acetone, dried, and their weight determined. The cleaning cycle was concluded when the final weight of the samples remained constant (3% variation in weight). Additionally, the removal of corrosion products was corroborated by observation under an optical microscope. The corrosion products collected were analyzed by X-ray diffraction.

The inhibition efficiency of lanthanum compounds was determined according to the following expression:

(2) $$I\left(\%\right)=\frac{W_{1}-W_{2}}{W_{1}}*100,$$

where *I* is the inhibition efficiency (%), *W*_1_ is the mass loss without inhibitor, and *W*_2_ is the mass loss with inhibitor (mg/cm^2^), both after 100 h of testing.

## 3. Results and Discussion

### 3.1. Mass Loss Tests

**Figure 1:**
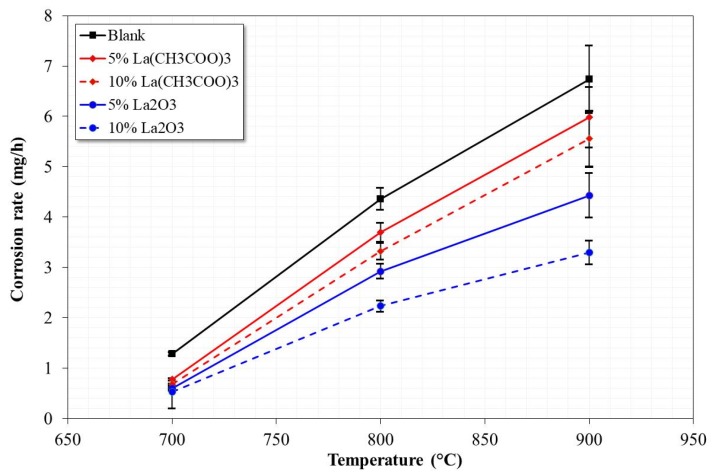
Corrosion rate for 304H steel after 100 h of testing in NaVO_3_ with and without the addition of La-base inhibitor at 700, 800, and 900 °C.

Figure 1 shows the loss of mass experienced by 304H steel after 100 h in NaVO_3_ with and without the addition of lanthanum compounds at three different working temperatures.

**Figure 2:**
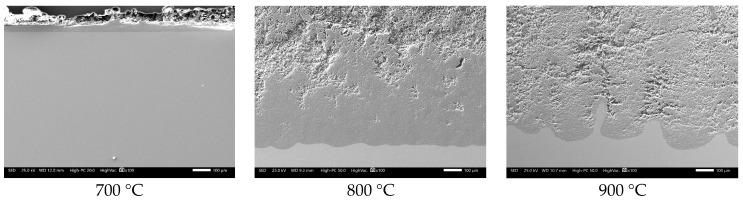
Cross-section aspect of 304H steel after corrosion test for 100 h in NaVO_3_.

It is observed that in the absence of the inhibitor, the steel showed the highest corrosion rate. The trend shows a constant increase in the corrosion rate as the temperature increases. This indicates that in the presence of V salts, 304H steel is not able to develop a stable protective oxide on its surface, as seen in Figure 2. The heavy corrosion-product layer formed indicates that the steel suffered an accelerated corrosion process. This is more evident when observing the surface profile; it was more irregular when the test temperature increased.

**Figure 3:**
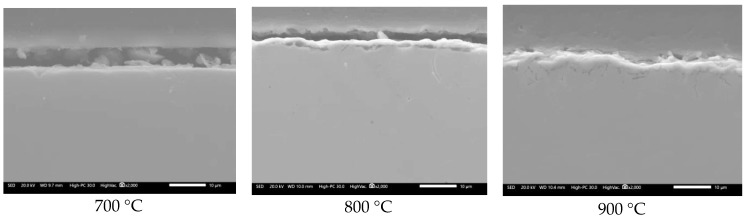
Cross-section aspect of 304H steel after 100 h of oxidation in air.

The foregoing contrasts with that shown in Figure 3, where it is observed that in the absence of V salts, the steel is capable of developing a thin film of protective oxide with a thickness close to 4 µm at 900 °C.

**Figure 4:**
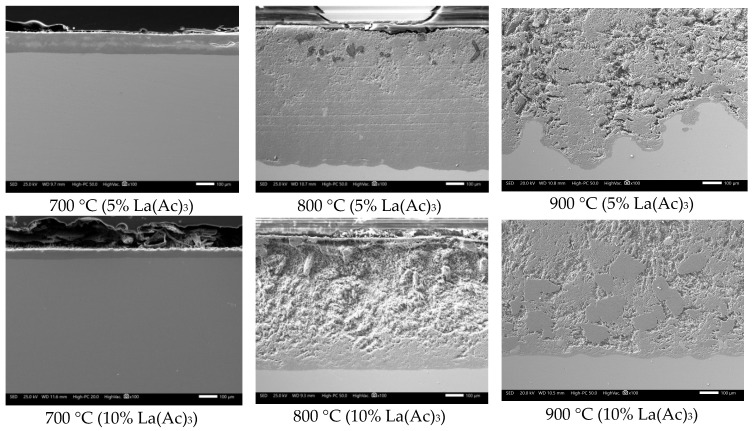
Cross-sectional aspect of 304H steel after corrosion testing for 100 h in NaVO_3_ with the addition of lanthanum acetate.

However, in the presence of La compounds, the corrosion rate of 304H steel tends to decrease depending on the type of La compound, its concentration, and test temperature (Figure 1). With the addition of lanthanum acetate, a lower reduction in the corrosion rate of the steel was obtained. In general, it was observed that the thickness of the corrosion-product layer tended to decrease, and the surface profile of the steel was more homogeneous (Figure 4). The above is more evident with the addition of 10% lanthanum acetate. However, in all cases, it was not possible to define the development of a stable protective oxide on its surface.

**Figure 5:**
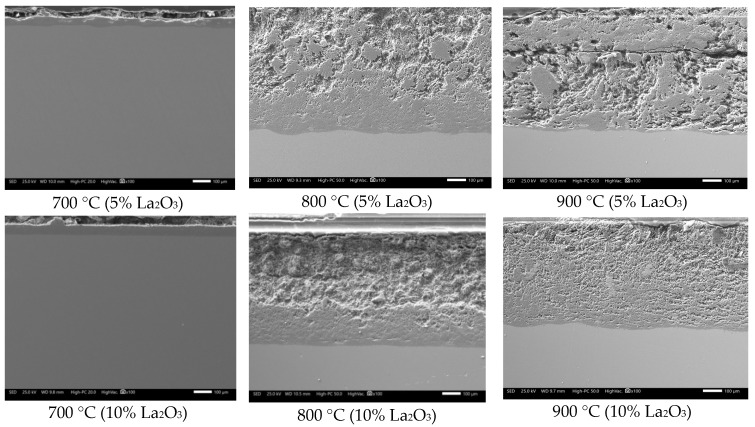
Cross-section appearance of 304H steel after corrosion test for 100 h in NaVO_3_ with the addition of La_2_O_3_.

On the other hand, with the addition of La_2_O_3_, the greatest reduction in the corrosion rate was obtained, and this effect is more visible at 800 and 900 °C (Figure 1). With the addition of lanthanum oxide (Figure 5), a smaller thickness of the corrosion-product layer was observed, in addition to a more homogeneous surface profile. Despite the decrease in the corrosion rate of steel, the development of a stable protective oxide on its surface was also not evident.

**Figure 6:**
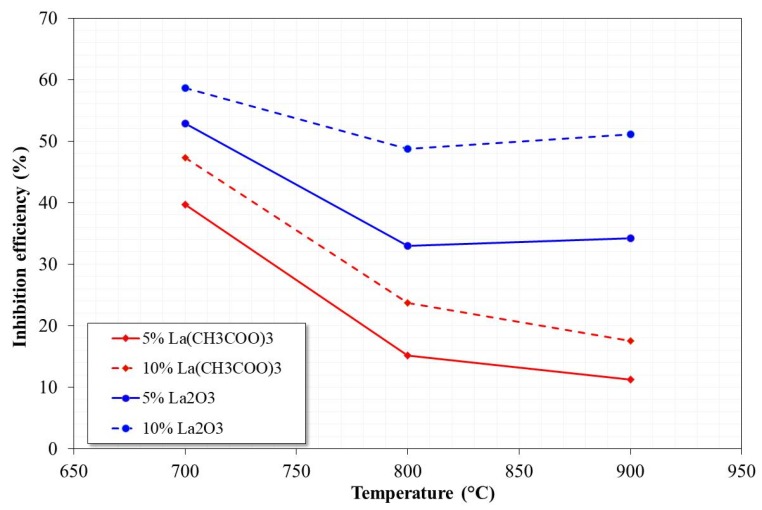
Inhibition efficiency of La compounds after 100 h of testing in NaVO_3_ at 700, 800, and 900 °C.

Figure 6 shows the inhibition efficiencies obtained by the addition of the La compounds. It is observed that the inhibition efficiency is a function of both the type of La compound added and its concentration. In general, inhibition efficiency increases by increasing the inhibitor concentration. On the other hand, the inhibition efficiency decreases by increasing the temperature in the case of lanthanum acetate, and in the case of lanthanum oxide it remains practically constant from 800 °C.

The above may be associated with the amount of V–La compounds formed. That is, the greater the amount of V–La compounds formed, the lower is the amount of free V available to react. Additionally, the barrier effect for the free diffusion of V compounds in the molten state is greater. In the most favorable case (10% La_2_O_3_), inhibition efficiencies between 50% and 60% were observed at the three test temperatures.

**Figure 7:**
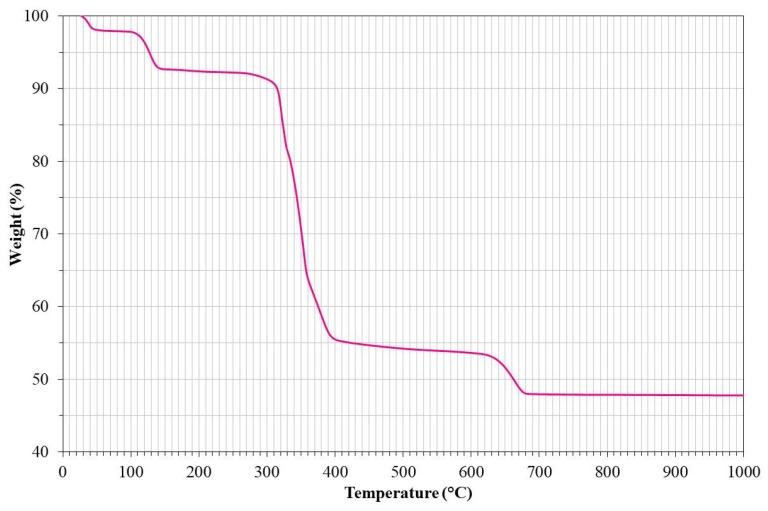
TGA (thermogravimetric analysis) curve of lanthanum acetate in air at a scanning rate of 5 °C/min.

The notable differences in the inhibition efficiency between lanthanum acetate and lanthanum oxide are due to the La content in both compounds. For a better understanding of this, Figure 7 shows the TGA (thermogravimetric analysis) curve of lanthanum acetate. From this figure, it can be seen that at the test temperatures of this study, lanthanum acetate decomposes to La_2_O_3_, experiencing a mass loss of approximately 52%.

According to the thermogram, it can be assumed that during the heating of the corrosive mixture, lanthanum acetate experienced a mass loss at temperatures below 200 °C due to moisture loss according to the following reactions [16]:

(3) $$La{\left(CH_{3}COO\right)}_{3}\cdot 1.5H_{2}O\to La{\left(CH_{3}COO\right)}_{3}\cdot H_{2}O+0.5H_{2}O,$$

(4) $$La{\left(CH_{3}COO\right)}_{3}\cdot H_{2}O\to La{\left(CH_{3}COO\right)}_{3}+H_{2}O.$$

Subsequently, around 350 °C experiences the greatest loss of mass (≈ 35%) due to the decomposition of acetate anions according to [17]:

(5) $$2La{\left(CH_{3}COO\right)}_{3}\to La_{2}{\left(CO_{3}\right)}_{3}+3CH_{3}COCH_{3},$$

(6) $$La_{2}{\left(CO_{3}\right)}_{3}\to La_{2}O_{2}CO_{3}+2CO_{2}.$$

Finally, the decomposition of the lanthanum oxycarbonate formed occurs around 640 °C, with an additional mass loss of approximately 5%, according to the following reaction [16,17]:

(7) $$La_{2}O_{2}CO_{3}\to La_{2}O_{3}+CO_{2}.$$

Then, this analysis allows us to establish that under the test conditions, lanthanum acetate was actually present as La_2_O_3_, and that finally, the corrosive mixtures with lanthanum acetate addition had an approximate content of 2.5% and 5.0% lanthanum oxide. Although the addition of the lanthanum compounds causes a slight decrease in the amount of NaVO_3_ available per unit area, it is considered that the aggressiveness of the salt is not affected. Therefore, it is considered that the decrease in aggressiveness of the corrosive mixture is only attributable to the presence of La_2_O_3_ and its interaction with vanadium species.

### 3.2. SEM Analysis

For simplicity and given that the characteristics observed at all test temperatures were similar, the analysis of the corroded samples at 900 °C will be discussed (in the absence and presence of an inhibitor at the highest concentration).

**Figure 8:**
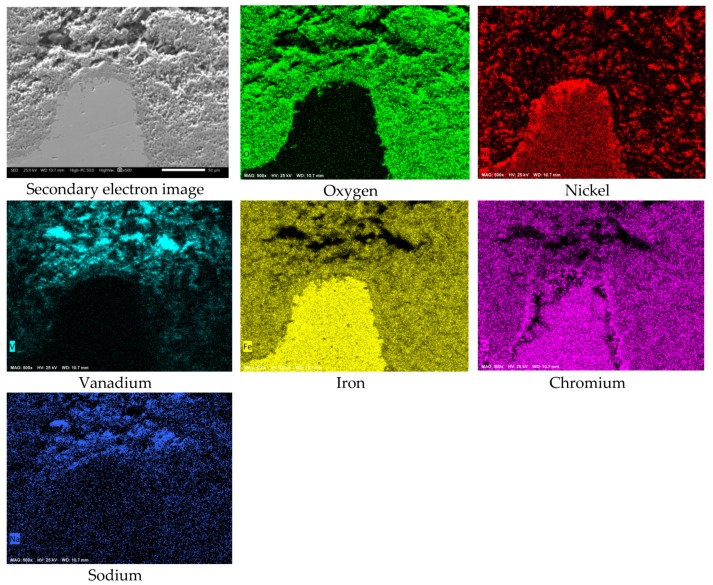
Cross-sectional aspect and element mapping of the 304H steel interface corroded in NaVO_3_ at 900 °C after 100 h.

**Figure 9:**
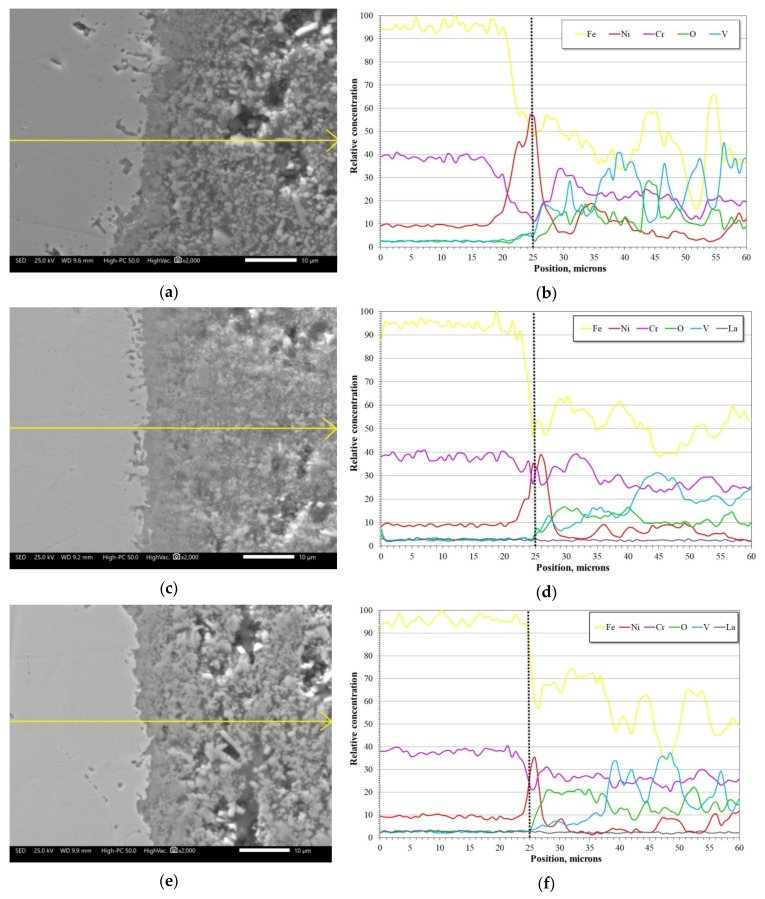
Line-scan of the relative concentration of elements for the 304H steel corroded at 900 °C for 100 h in: (**a**,**b**) NaVO_3_, (**c**,**d**) NaVO_3_-10%La(Ac)_3_, (**e**,**f**) NaVO_3_-10%La_2_O_3_. The dotted vertical line indicates the metal/corrosion products interface.

Figure 8 shows the cross-sectional aspect and element mapping of the metal–corrosion products interface of 304H steel corroded in NaVO_3_ at 900 °C for 100 h. Cr and O mapping allows us to define the presence of a thin discontinuous film of chromium oxide on the metal surface. However, it is also observed that the steel shows both Cr and Fe depletion and Ni enrichment in the metal surface. An elements line-scan through the interface (Figure 9a,b) allows the defining of the above; in addition, there is a region near 10 microns enriched in Ni and with a depletion in both Cr and Fe in the surface of the steel. This phenomenon is common in active corrosion processes when the material is unable to develop a stable protective oxide due to its continuous dissolution by the action of the molten salts [18,19].

Although it has been observed that pure Cr is capable of developing a protective oxide in the presence of NaVO_3_ at 900 °C [6], the protective characteristics of Cr-based oxides developed onto alloys may be different. This is due to the simultaneous formation of other metal oxides, of the alloy elements, which causes a reduction in the protective properties of the protective oxide. Several studies by Rapp et al. [5,20,21], have shown that the coexistence of different metal oxides enhance their synergistic dissolution.

On the other hand, the mapping of Ni and O allows us to observe a superficial enrichment in Ni, as well as a lower density, as a corrosion product. Previous studies have shown that pure Ni shows good corrosion resistance in NaVO_3_ at 900 °C [14], being able to develop a protective oxide (NiO) and highly stable corrosion products (Ni vanadates). Moreover, according to the element mapping, it is observed that the corrosion-product layer is formed by metal oxides and vanadates (of the alloy elements of the steel), and in a lower concentration by vanadium salts.

**Figure 10:**
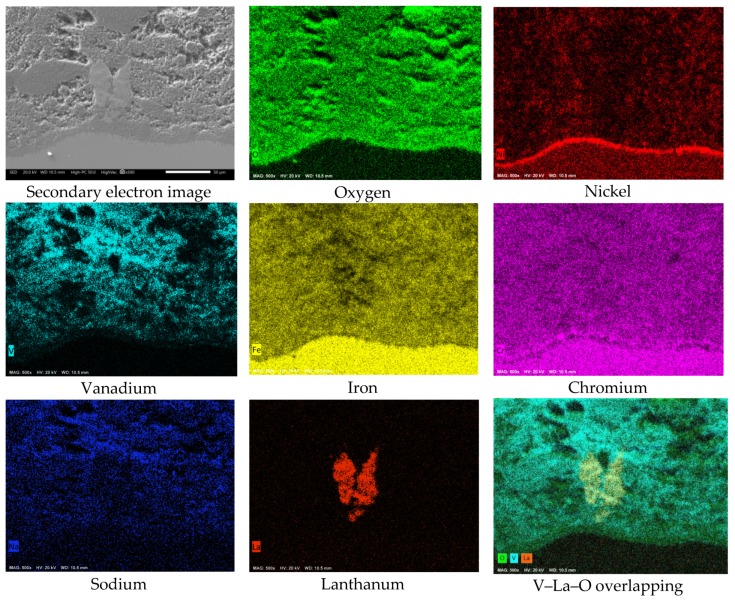
Cross-sectional aspect and element mapping of the 304H steel interface corroded in NaVO_3_ with 10% La(Ac)_3_ at 900 °C after 100 h.

Figure 10 shows the cross-sectional aspect and element mapping of the metal–corrosion products interface of 304H steel corroded in the NaVO_3_-10%La(Ac)_3_ mixture at 900 °C for 100 h. The main difference that can be observed is the surface profile of the steel. It is notable that in the presence of La(Ac)_3_, the degradation experienced by the steel was lower. However, although the Cr and O mapping makes it possible to define the presence of a thin discontinuous film of chromium oxide on the metal surface, it can be seen that it has greater continuity than that observed in the absence of the inhibitor (Figure 8). Similarly, there is a lower Cr depletion in the alloy and an apparent greater Ni enrichment on the metal surface. Figure 9c,d shows that the depletion zone in Cr and Fe was smaller (approximately 5 µm) than that observed in the absence of the inhibitor. In addition, the Ni-enriched zone is found both in the alloy and on the metal surface. This indicates the presence of a Ni-rich surface layer. The formation of a Ni-rich layer on the metal surface may be due to a decrease in the aggressiveness of molten salts.

The element mapping also makes it visible that the corrosion-product layer consists of metal oxides, metal vanadates, and a greater amount of vanadium salts than that observed in the absence of the inhibitor (Figure 8). According to the V, La, and O mapping, the presence of new compounds possibly of the La–vanadate type is evident. These phases have a size of around 50 µm and are observed near the metal surface. The above characteristics allow us to infer that the addition of the La-based inhibitor decreased the aggressiveness of NaVO_3_ either due to the formation of V–La–O compounds and/or new V compounds with less aggressiveness.

**Figure 11:**
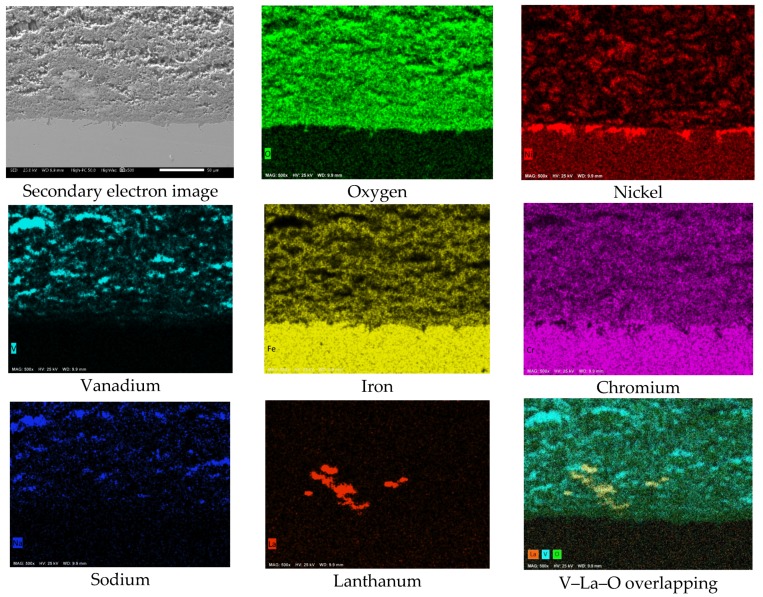
Cross-sectional aspect and element mapping of the 304H steel interface corroded in NaVO_3_ with 10% La_2_O_3_ at 900 °C after 100 h.

**Figure 12:**
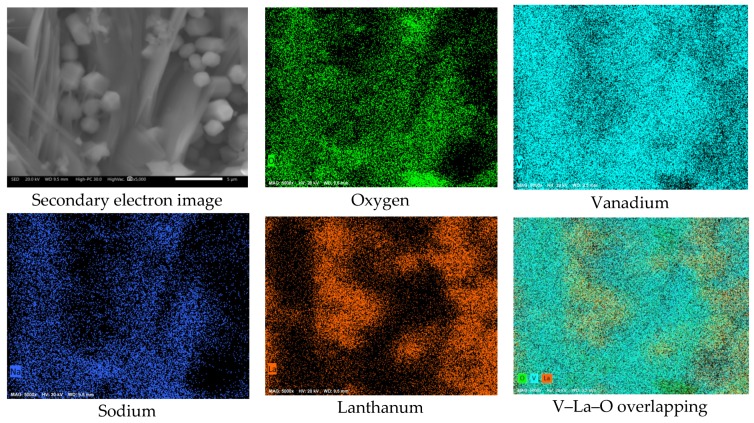
Approach to the corrosion-product layer and elements mapping of the 304H steel corroded in NaVO_3_ with 10% La_2_O_3_ at 900 °C after 100 h.

Figure 11 shows the cross-sectional aspect and element mapping of the metal–corrosion products interface of 304H steel corroded in the mixture NaVO_3_-10%La_2_O_3_ at 900 °C for 100 h. In general, the observed characteristics are similar to those mentioned with the addition of 10% La(Ac)_3_ (Figure 10). The main difference is the presence of a more homogeneous surface profile. According to Figure 9e,f, it is observed that the zone of depletion in Cr and Fe and enrichment in Ni was smaller (approximately 2 µm) than that observed with the addition of La acetate. Based on the above, it can be inferred that by increasing the La^3+^ concentration, the melt corrosivity decreases due to the formation of V–La–O compounds and/or new V compounds with less aggressiveness. These results indicate that in the absence of the inhibitor, the vanadium salts continually attacked the chromium and iron oxides, thereby causing the surface Ni-enrichment observed in the alloy. However, in the presence of inhibitor, this enrichment tended to decrease and was lower with the addition of La_2_O_3_. This suggests that the aggressiveness of the melt decreased due to the formation of new V–La–O phases that stabilized the available free vanadium, thus reducing its availability for the formation of new unstable metal vanadates. An approach to the corrosion-product layer makes it possible to define the presence of small V–La–O crystals, smaller than 5 µm (Figure 12), surrounded by vanadium salts (V–Na–O).

**Figure 13:**
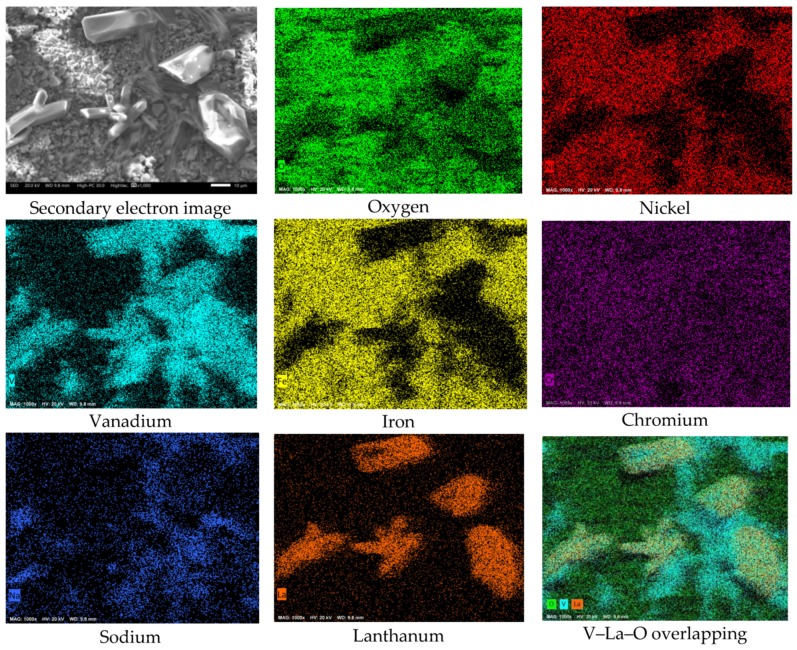
Surface view of the corrosion-product layer and element mapping of the 304H steel corroded in NaVO_3_ with 10% La_2_O_3_ at 900 °C after 100 h.

On the other hand, a superficial analysis of the corrosion-product layer (Figure 13) allowed us to observe the presence of V–La–O crystals with a size greater than 50 µm. This may be the reason why in Figure 10 and Figure 11, a large number of V–La–O particles were not observed near the metal surface. It is possible that during growth, these highly stable phases were displaced towards the surface of the corrosion-product layer. Similarly, the high density of Ni was possibly noticeable in the form of vanadate. Of the possible metallic vanadates formed, those based on La or Ni have a melting point greater than that of the Fe and Cr vanadates.

### 3.3. XRD Analysis

**Figure 14:**
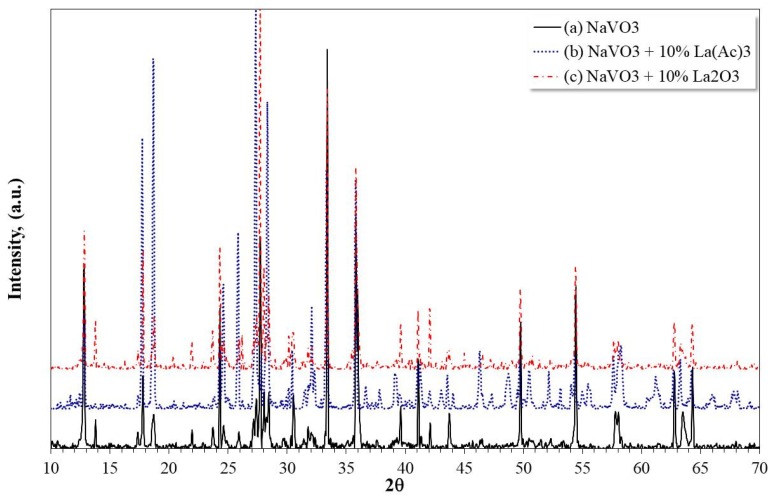
X-ray diffraction patterns of corrosion products formed on 304H steel corroded at 900 °C for 100 h in: (**a**) NaVO_3_, (**b**) NaVO_3_-10% La(Ac)_3_, and (**c**) NaVO_3_-10% La_2_O_3_.

In order to determine the type of compounds formed, Figure 14 shows the X-ray diffractograms of corrosion products formed on 304H steel corroded at 900 °C for 100 h with and without the addition of La compounds. Because the X-ray diffractograms of corrosion products at 700 and 800 °C are similar, only that obtained at 900 °C is shown.

The identification of the compounds indicated that in the absence of a corrosion inhibitor, the main corrosion products detected were mainly oxides and spinels (Cr_2_O_3_, Fe_2_O_3_, NiO, FeCr_2_O_4_, NiFe_2_O_4_, NiCr_2_O_4_), metallic vanadates (CrVO_4_, FeVO_4_, Ni_3_(VO_4_)_3_), in addition to NaVO_3_. The proportion of them varied with the test temperature; the presence of oxides and spinels was greater as the test temperature increased. On the other hand, in the presence of the inhibitor and regardless of the type of La compound, in addition to the above compounds, the presence of La vanadate (LaVO_4_) was detected.

Based on the above, it can be assumed that the process of degradation of the metal elements of the alloy began according to the following reactions [22]:

(8) $$2Fe^{3+}+3O^{2-}\to Fe_{2}O_{3},$$

(9) $$2Cr^{3+}+3O^{2-}\to Cr_{2}O_{3},$$

(10) $$Ni^{2+}+O^{2-}\to NiO.$$

This caused the development of a Cr-rich protective oxide, but with the simultaneous growth of both Fe and Ni oxides. Subsequently, the synergistic dissolution of the protective layer occurred by the action of molten NaVO_3_, resulting in the formation of metallic vanadates according to the following reactions [23]:

(11) $$Fe_{2}O_{3}+2NaVO_{3}\to 2FeVO_{4}+Na_{2}O,$$

(12) $$Cr_{2}O_{3}+2NaVO_{3}\to 2CrVO_{4}+Na_{2}O,$$

(13) $$3NiO+2NaVO_{3}\to Ni_{3}{\left(VO_{4}\right)}_{2}+Na_{2}O.$$

The dissolution of the oxides causes an increase in the melt basicity, and therefore, the formation of sodium orthovanadate is possible according to the following reaction [23]:

(14) $$NaVO_{3}+Na_{2}O\to Na_{3}VO_{4}.$$

The formation of vanadium salts of greater basicity affects the charge transport due to the stabilization of the highest valence state of V affecting the V^5+^:V^4+^ relation [5].

**Figure 15:**
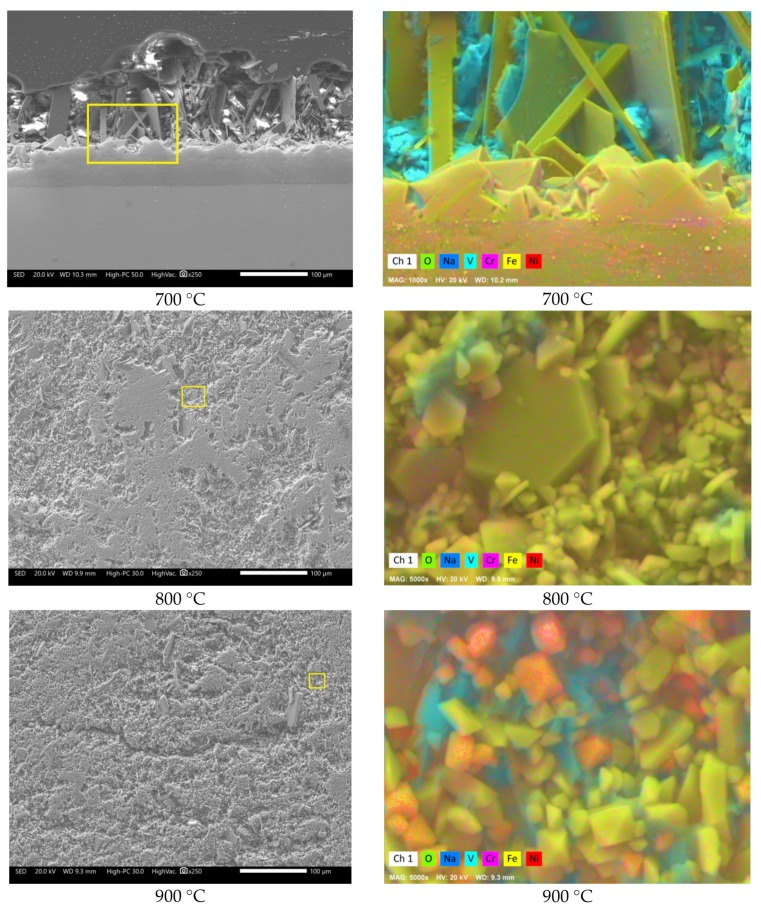
Approach to the corrosion-product layer and element mapping of 304H corroded steel in NaVO_3_ at 700, 800, and 900 °C after 100 h. The box indicates the area where the element mapping was performed.

The formation of these metallic vanadates results in compounds with melting points greater than 850 °C; however, in all cases, an analysis of the corrosion products away from the metallic surface shows the presence of Fe–Cr–Ni–O compounds and a low presence of V–Na–O compounds, as seen in Figure 15.

The presence of well-defined crystalline forms associated with Fe–Cr–Ni–O compounds may correspond to the spinels detected by X-ray diffraction. However, their presence at all test temperatures and in sites away from the metallic surface does not correspond to the detachment of oxides from the protective layer. This may be due to a more complex mechanism of reprecipitation of oxides.

In order to justify the presence of these crystalline phases, an understanding of the stability of the phases present in the corrosion products is necessary. In this sense, and according to studies by Walczak and Filipek [24], it is known that CrVO_4_ in liquid state (860 °C) decomposes, causing the precipitation of Cr_2_O_3_. Similar results have been reported by Kerby and Wilson [25]; they have indicated that in basic vanadium salts, the Cr and Fe vanadates decompose into their respective oxides.

The above justifies, in some way, the presence of the metallic oxides detected, as long as the temperature is higher than the melting points of each metallic vanadate (> 850 °C). However, the massive presence of Fe–Cr–Ni–O spinels in areas far from the metal surface may be due to other mechanisms.

In this sense, it has been reported that despite the fact that metal vanadates have melting points greater than 800 °C, their stability depends on the presence of other V salts in the liquid state. It has been reported that the presence of metallic vanadates and vanadium salts may form eutectic mixtures with melting points below 700 °C [24,25]. In addition, as the partial pressure of oxygen increases, then the eutectic mixture undergoes cooling due to an attempt to stabilize its oxygen deficiency at low partial oxygen pressures [25].

The above suggests that the molten vanadium salts can dissolve the metallic vanadates (Fe, Cr, and Ni) at temperatures below 700 °C, and since sodium-rich vanadium salts are ionic conductors [26], the presence of the crystalline forms associated with spinels and/or oxides in areas away from the metal surface (higher partial oxygen pressure) can be due to the simultaneous reprecipitation of the metal cations according to the following reactions:

(15) $$FeVO_{4}+2CrVO_{4}+O^{2-}\to FeCr_{2}O_{4}+3VO_{3}^{-},$$

(16) $$Ni_{3}{\left(VO_{4}\right)}_{2}+6FeVO_{4}+4O^{2-}\to 3NiFe_{2}O_{4}+8VO_{3}^{-},$$

(17) $$Ni_{3}{\left(VO_{4}\right)}_{2}+6CrVO_{4}+4O^{2-}\to 3NiCr_{2}O_{4}+8VO_{3}^{-},$$

(18) $$2FeVO_{4}+O^{2-}\to Fe_{2}O_{3}+2VO_{3}^{-},$$

(19) $$2CrVO_{4}+O^{2-}\to Cr_{2}O_{3}+2VO_{3}^{-},$$

(20) $$Ni_{3}{\left(VO_{4}\right)}_{2}+O^{2-}\to 3NiO+2VO_{3}^{-}.$$

In addition, the new phases formed (spinels) are more stable due to their high melting points: 2000 °C for FeCr_2_O_4_ [27], 1660 °C for NiFe_2_O_4_ [28], and 2083 °C for NiCr_2_O_4_ [29]. The above can lead to the regeneration of NaVO_3_, thus causing an active corrosion process.

(21) $$2VO_{3}^{-}+Na_{2}O↔2NaVO_{3}+O^{2-}$$

On the other hand, in the presence of the inhibitor in addition to the above reactions, La_2_O_3_ reacts with the vanadium salts, thus forming the La vanadate (LaVO_4_) according to the following reaction:

(22) $$2NaVO_{3}+La_{2}O_{3}\to 2LaVO_{4}+Na_{2}O.$$

Through this reaction, lanthanum oxide stabilizes the vanadium cation and reduces the ionic exchange that favors the dissolution process of the protective layer [7,9] by reducing the acid character of the melt according to the following reaction [9,10]:

(23) $$VO_{3}^{-}+O^{2-}↔VO_{4}^{3-}.$$

Several studies have shown that rare earths in the presence of vanadium salts form stable phases [30,31,32] and are also responsible for the degradation of thermal barrier coatings [33]. However, their use as a potential corrosion inhibitor has been demonstrated through the results of this study. Lanthanum compounds decrease the aggressiveness of the melt because they neutralize the V due to the formation of lanthanum orthovanadate, which has a melting point greater than 1800 °C [34]. Additionally, it also increase the melt basicity and, therefore, the corrosivity of the vanadium salts decreases [5,6,14].

## 4. Conclusions

Through the results obtained from this study, it can be established that the rare-earth-based compounds (La(Ac)_3_, La_2_O_3_) act as inhibitors of corrosion by vanadium salts. Because lanthanum acetate decomposes in lanthanum oxide from 640 °C, in all cases, the compound that acted as a corrosion inhibitor was La_2_O_3_. At the maximum concentration of inhibitor evaluated (10% La_2_O_3_), a reduction in corrosion rate from 50%–60% was obtained at all test temperatures. Lanthanum oxide reacted with the vanadium salts, forming a new V–La–O compound (LaVO_4_) with a melting point greater than 1800 °C. Its melting point is much higher than those of other refractory vanadates (Mg_3_V_2_O_8_ = 1200 °C, Ni_3_V_2_O_8_ = 1310 °C) formed when magnesium or nickel compounds are used as corrosion inhibitors. In this way, the corrosive action of vanadium was reduced by decreasing its availability, favoring the formation of vanadium salts of greater basicity, stabilizing the highest valence state of V, reducing the charge transport by affecting the V^5+^:V^4+^ ratio, and reducing ionic transport by increasing the basic character of the melt.

## Figures and Tables

**Table 1:** Main vanadium compounds formed according to Equation (1).

x/y Ratio	Reaction Products	Vanadium Salt	Melting Point
3 (x = 3; y = 1)	$3Na_{2}O\cdot V_{2}O_{5}+3SO_{3}$	Na_3_VO_4_ (Sodium Orthovanadate)	850 °C
2 (x = 2; y = 1)	$2Na_{2}O\cdot V_{2}O_{5}+2SO_{3}$	Na_4_V_2_O_7_ (Sodium Pyrovanadate)	640 °C
1 (x = 1; y = 1)	$Na_{2}O\cdot V_{2}O_{5}+SO_{3}$	NaVO_3_ (Sodium Metavanadate)	630 °C
0.167 (x = 1; y = 6)	$Na_{2}O\cdot 6V_{2}O_{5}+SO_{3}$	Na_2_O·V_2_O_4_·5V_2_O_5_ (β-sodium vanadyl vanadate)	625 °C
0.417 (x = 5; y = 2)	$5Na_{2}O\cdot 12V_{2}O_{5}+5SO_{3}$	5Na_2_O·V_2_O_4_·11V_2_O_5_ (γ-sodium vanadyl vanadate)	535 °C

## References

[1] Wong-Moreno A., Mujica Martinez Y., Martinez L. (1994). High temperature corrosion enhanced by residual fuel oil ash deposits. CORROSION/94.

[2] Gonzalez-Rodriguez J.G., Haro S., Martinez-Villafañe A., Salinas-Bravo V.M., Porcayo-Calderon J. (2006). Corrosion performance of heat resistant alloys in Na_2_SO_4_-V_2_O_5_ molten salts. Mater. Sci. Eng. A.

[3] Zheng X., Rapp R.A. (1995). Electrochemical impedance study of platinum electrode in fused Na_2_SO_4_-10 mole percent NaVO_3_ melts. J. Electrochem. Soc..

[4] Otsuka N., Rapp R.A. (1990). Effects of chromate and vanadate anions on the hot corrosion of preoxidized Ni by a thin fused Na_2_SO_4_ film at 900 °C. J. Electrochem. Soc..

[5] Hwang Y.-S., Rapp R.A. (1989). Termochemistry and solubilities of oxides in sodiun sulfate-vanadate solutions. Corrosion.

[6] Porcayo-Calderon J., Salinas-Bravo V.M., Rodriguez-Diaz R.A., Martinez-Gomez L. (2015). Effect of the NaVO_3_-V_2_O_5_ ratio on the high temperature corrosion of chromium. Int. J. Electrochem. Sci..

[7] Montero X., Galetz M.C. (2016). Inhibitors and coatings against vanadate-containing oil ash corrosion of boilers. Surf. Coat. Technol..

[8] Kerahroodi M.S.A., Rahmani K., Yousefi M. (2018). The inhibitory effect of magnesium sulfonate as a fuel additive on hot corrosion of generating tubes of power plant boiler. Oxid. Met..

[9] Rocca E., Aranda L., Moliere M., Steinmetz P. (2002). Nickel oxide as a new inhibitor of vanadium-induced hot corrosion of Superalloys - comparison to MgO-based inhibitor. J. Mater. Chem..

[10] Di Salvia N., Malavasi M.P., Di Salvia G., Vaccari A. (2015). formation of Ni and Mg vanadates during the flameless oxy-combustion of heavy fuels. Fuel Process. Technol..

[11] Rocca E., Aranda L., Molière M. (2008). Chemistry of ash-deposits on gas turbines hot parts: Reactivity of nickel, zinc and iron oxides in (Na, V, S) molten salts. Mater. Sci. Forum.

[12] Rhys-Jones T.N., Nicholls J.R., Hancock P. (1983). Effects of SO_2_/SO_2_ on the efficiency with which MgO inhibits vanadic corrosion in residual fuel fired gas turbines. Corrosion Sci..

[13] Hancock P. (1987). Vanadic and chloride attack of superalloys. Mater. Sci. Technol..

[14] Porcayo-Calderon J., Ramos-Hernandez J.J., Mayén J., Porcayo-Palafox E., Pedraza-Basulto G.K., Gonzalez-Rodriguez J.G., Martinez-Gomez L. (2017). High temperature corrosion of nickel in NaVO_3_-V_2_O_5_ Melts. Adv. Mater. Sci. Eng..

[15] Sotelo-Mazón O., Porcayo-Calderon J., Cuevas-Arteaga C., Ramos-Hernandez J.J., Ascencio-Gutierrez J.A., Martinez-Gomez L. (2014). EIS Evaluation of Fe, Cr, and Ni in NaVO_3_ at 700 °C. J. Spectrosc..

[16] Rizzo da Rocha S.M., da Silva Queiroz C.A., Abrão A. (2002). Synthesis and characterization of lanthanum acetate for application as a catalyst. J. Alloy. Compd..

[17] Hussein G.A.M. (1994). Spectrothermal investigation of the decomposition course of lanthanum acetate hydrate. J. Therm. Anal..

[18] Salinas G., Gonzalez-Rodriguez J.G., Porcayo-Calderon J., Salinas-Bravo V.M., Espinosa-Medina M.A. (2012). Effect of minor alloying elements on the corrosion behavior of Fe-40Al in NaCl-KCl molten salts. Int. J. Corrosion.

[19] Salinas-Solano G., Porcayo-Calderon J., Gonzalez-Rodriguez J.G., Salinas-Bravo V.M., Ascencio-Gutierrez J.A., Martinez-Gomez L. (2014). High temperature corrosion of inconel 600 in NaCl-KCl molten salts. Adv. Mater. Sci. Eng..

[20] Rapp R.A. (2002). Hot corrosion of materials: A fluxing mechanism?. Corrosion Sci..

[21] Hwang Y.-S., Rapp R.A. (1990). Synergistic dissolution of oxides in molten sodium sulfate. J. Electrochem. Soc..

[22] Espinosa-Medina M.A., Carbajal-De la Torre G., Liu H.B., Martínez-Villafañe A., González-Rodriguez J.G. (2009). Hot corrosion behaviour of Fe-Al based intermetallic in molten NaVO_3_ salt. Corrosion Sci..

[23] Longa-Nava Y., Zhang Y.S., Takemoto M., Rapp R.A. (1996). Hot corrosion of nickel-chromium and nickel-chromium-aluminum thermal-spray coatings by sodium sulfate-sodium metavanadate salt. Corrosion.

[24] Walczak J., Filipek E. (1989). Phase equilibria in the V_2_O_5_-Cr_2_O_3_ System. J. Therm. Anal..

[25] Kerby R.C., Wilson J.R. (1973). Solid-liquid phase equilibria for the ternary systems V_2_O_5_-Na_2_O-Fe_2_O_3_, V_2_O_5_-Na_2_O-Cr_2_O_3_, and V_2_O_5_-Na_2_O-MgO. Can. J. Chem..

[26] Rost E. (1985). Reduction of Fused Sodium Series A. Physical and inorganic chemistry. Vanadates Acta chem..

[27] Huang W., Chen M., Shen X., Shan Y., He M., Wang N. (2016). Viscosity property and Raman spectroscopy of FeO-SiO_2_-V_2_O_3_-TiO_2_-Cr_2_O_3_ slags. in advances in molten slags. Proceedings of the 10th International Conference on Molten Slags, Fluxes and Salts 2016.

[28] Tonkov E.I., Tonkov E.Y. (1992). High Pressure Phase Transformations: A Handbook.

[29] Kjellqvist L., Selleby M., Sundman B. (2008). Thermodynamic modelling of the Cr–Fe–Ni–O system. Calphad.

[30] Jones R.L. (1991). Oxide acid-base reactions relating to the inhibition of vanadium attack on REY zeolite catalysts. J. Catal..

[31] Reidy R.F., Jones R.L. (1995). Thermogravimetric analysis of the reaction of CeO_2_ with the NaVO_3_-SO_3_ system. J. Electrochem. Soc..

[32] Zhang Y.S., Rapp R.A. (1987). Solubilities of CeO_2_, HfO_2_ and Y_2_O_3_ in fused Na_2_SO_4_-30 mol% NaVO_3_ and CeO_2_ in pure Na_2_SO_4_ at 900 °C. Corrosion.

[33] Jones R.L. (1997). Some aspects of the hot corrosion of thermal barrier coatings. J. Therm. Spray Technol..

[34] Kamada T., Kaneko T., Daimon K., Hikichi Y., Ota T. Synthesis and properties of monazite type LaVO_4_. Proceedings of the Preprints of Annual Meeting of the Ceramic Society of Japan.

